# Quality changes in workplace health promotion over time: an extended validation of quality criteria from a longitudinal perspective

**DOI:** 10.1007/s10389-023-01956-8

**Published:** 2023-06-06

**Authors:** Gert Lang, Paulino Jiménez

**Affiliations:** 1 Austrian Health Promotion Fund, Austrian National Public Health Institute, Vienna, Austria; 2 Institute of Psychology, University of Graz, Graz, Austria

**Keywords:** Workplace health promotion, quality assurance, confirmatory factor analysis, validity, reliability

## Abstract

**Aim:**

The quality of workplace health promotion (WHP) is essential for the successful deployment of financial and human resources. The aim of this paper is to evaluate the measurement quality of a WHP instrument with 15 quality criteria over time. In addition, it examines whether the quality of WHP in the enterprises concerned changed over time and whether typical developments can be identified. Finally, the role of company parameters such as size and implementation phase are analysed in relation to how WHP develops over time.

**Subjects and methods:**

Evaluations of the quality of WHP collected between 2014 and 2021 were available at two and three measurement points for 570 and 279 enterprises, respectively. To assess the longitudinal measurement structure, confirmatory factor analyses were carried out followed by structural equation modelling to analyse causality. Cluster analysis was used to identify typical developments, and differences in company parameters were analysed with MANOVA.

**Results:**

The results prove that the 15 quality criteria can be used to evaluate the quality of WHP in enterprises in a valid and reliable manner, both cross-sectionally and longitudinally. The quality of WHP in the enterprises concerned remained relatively stable over approximately 12 years. The cluster solution revealed three different developments characterized by increasing, stable or decreasing quality.

**Conclusion:**

Measurements using a quality evaluation system permit a good assessment of WHP in enterprises. WHP quality also depends on company parameters; here more support should be provided to motivate enterprises in the long run, particularly in the sustainability phase.

## Introduction

Health promotion can be seen as the standard in the canon of health and preventive measures. Health promotion in company settings is additionally of great importance because it can reach a large part of the population, thus contributing to health and well-being (Kuhn and Chu 2022; Naidoo and Wills 2016). In other words, workplace health promotion (WHP) represents a unique opportunity to change health behaviour at work and provides an ideal environment for achieving better health. As such measures are associated with great efforts on the part of enterprises, both in financial terms and in connection with the commitment of internal project managers, the quality of WHP is all the more important. Therefore, it is essential to investigate whether and how quality can change on different levels and what influences these changes.

Great importance has been attached to WHP, particularly in relation to current challenges such as the COVID-19 pandemic, the energy, economic and climate crises, shifting demographics, digitalization and the flexibilization of work, for example, and it has the potential to maintain or even increase individual capacity to work and the supply of labour. WHP denotes a strategy for action comprising several levels of analysis and implementation (employees, organization, work) and aims to boost health resources in the context of work. WHP is essentially understood as a long-term development strategy for companies and organizations and pursues the goal of improving health at work, increasing the well-being of employees and preventing illness (Burton 2010; Faller 2017).

The current state of research indicates that WHP can be assumed to have a positive effect on physical and mental health. Proof of this can be found, for example, in reviews of the literature on WHP in relation to increasing physical activity, managing stress, stopping smoking and improving musculoskeletal health (Siguero et al. 2021), as well as in a meta-analysis of randomized controlled trials of WHP in relation to self-perceived health and work absence due to sickness (Rongen et al. 2013). WHP programmes to promote health and well-being at work can be expected to be particularly effective when comprehensive (i.e. multimodal and holistic) person-related and work-related health promotion interventions are implemented (Daniels et al. 2022; Gerhardt et al. 2019; Pham et al. 2019).

To successfully implement WHP, it is recommended to take account of typical (facilitating or hindering) factors as well as those which are reported on frequently (Bauer et al. 2014; Rojatz et al. 2017). Working on multiple levels, these factors address structural (framework) conditions, requirements and opportunities on the one hand which make the process of implementing WHP, in some circumstances, more (or less) successful on the other. With regard to structural factors, reviews of the literature attest that the likelihood of implementing health-related interventions or promoting health and well-being in the workplace decreases in relation to the size of the company, in other words whether it is large, medium or small (Hollederer and Wießner 2015; McCoy et al. 2014). The size of an enterprise also affects factors on several levels, although in smaller enterprises, the barriers, beliefs and challenges tend to dominate (McCoy et al. 2014; Taylor et al. 2016). In comparison with larger companies, smaller enterprises often have less financial leeway and more frequently shy away from associated costs including investment risks, uncertain return on investments or programme costs (Gerhardt et al. 2019; McCoy et al. 2014; Taylor et al. 2016). In addition, smaller businesses are frequently affected by insufficient in-house expertise or practical capacity (Jessiman-Perreault et al. 2020) while some lack third-party support for health and well-being programmes in the workplace in rural settings, for example (McCoy et al. 2014). Implementation in small enterprises is often constrained by limited resources in the human resource area (Gerhardt et al. 2019), insufficient qualifications and knowledge (Jessiman-Perreault et al. 2020), lower acceptance of and interest in WHP (Taylor et al. 2016) or a lack of support at all levels of management (McCoy et al. 2014). This contrasts with a shorter list of potential advantages for smaller enterprises, such as lower bureaucratic hurdles, easier implementation, higher participation rates, more direct possibilities for incorporating employees’ suggestions, a greater potential for teamwork development and group bonding (McCoy et al. 2014).

In practical terms, companies usually tackle the promotion of health and well-being in different steps or phases of development (Bauer et al. 2014). Most enterprises start off by undertaking one-off or sporadic health- or work-oriented interventions. The essence of WHP lies in the combination of approaches and measures, however, which create adequate supportive conditions and structures in the workplace setting in connection with empowering individuals to follow a healthier lifestyle. In line with the guiding principles of the Luxembourg Declaration on Workplace Health Promotion, all members of staff should be involved in WHP (participation), WHP should be taken into account in all important decisions and in all areas of a company (integration), both individual-directed and environment-directed measures should be implemented, combining the strategy of risk reduction with the strategy of developing protection factors and health potentials (comprehensiveness) and all activities and methods should be implemented systematically (project management) (cf. ENWHP 2007).

The initial phase primarily serves to establish suitable structures and processes for WHP and usually takes one to three years depending on the goals and setting (e.g. the size of an enterprise). To stabilize WHP and establish it on an ongoing basis, usually several implementation cycles are required to optimize processes. In the spirit of organizational health development (Bauer and Jenny 2017), the sustainability phase is meant to put health-promoting structures and process on a permanent footing and to allow their gradual integration in the day-to-day operation of an enterprise with the goal of achieving systematic workplace health management. This can lead to the benefit of a continuous realization or sustainable effect of WHP that is then associated with a virtuous circle and the possibility of creating a health-promoting corporate culture (cf. Badura et al. 2016; Schein 2010). Some enterprises also refer to challenges (Goldgruber and Ahrens 2010; Robroek et al. 2009) such as keeping WHP attractive over time and the necessity of always setting new impulses.

Effective and sustainable WHP requires appropriate quality assurance and the application of suitable quality standards (processes, methods and measures). In line with the findings presented above, to evaluate the quality of WHP, it is appropriate to use the dimensions of quality proposed by Donabedian (1980) and expanded on by Kliche et al. (2004) consisting of structure, process, outcome and concept. Structural quality relates to the setting as well as the structural requirements and (framework) conditions (particularly on a contextual and organizational level), the quality of the process is about how implementation steps are carried out (e.g. project management, the Plan-Do-Check-Act cycle), the quality of the outcome describes the achieved effects, successes and monitoring of satisfaction (e.g. the evaluation of effectiveness and sustainability) and the quality of the concept is about selecting the most effective interventions according to the latest scientific findings. These dimensions are used in health promotion and disease prevention to deploy quality criteria in an objective, consistent and comparable manner. To this end, the Austrian Network for Workplace Health Promotion, for example, has defined 15 different quality criteria within the framework of its quality management (QM) system and employs them to award WHP quality certificates (BGF-Gütesiegel) for the certification of enterprises (Heigl 2014).

This QM system has not only proven its worth in practice but also fulfils scientific criteria. The quality indicators used are listed in Table 1. A more recent quantitative study (Lang et al. 2019) validated the quality criteria in accordance with criteria of measurement quality (content and construct validity, internal consistency) with the help of cross-sectional data from the QM system which also showed a good interrater reliability (ICC = 0.682). This study also proposed examining longitudinal changes in the quality of WHP implemented over a longer observation period. The 15 quality indicators of WHP can be represented as a general factor; at the same time the bi-factor model revealed that several residual factors lie behind it (Lang et al. 2019).

**Table 1:** The 15 quality indicators for WHP

No.	Description
1	Corporate principles/culture
2	Structure (of the project)
3	Responsibilities/contact persons
4	Target group orientation
5	Diagnostic phase/tools/needs assessment
6	Employee orientation
7	Communication
8	Environment-directed measures
9	Individual-directed measures
10	Management
11	Quality of the formulation/scope of the goals
12	Monitoring of results and evaluation
13	Attainment of goals
14	Sustainability
15	General evaluation

Source: Lang et al. (2019: 700)

Against the background of these considerations, this paper focuses on the following research questions: (1) How can the measurement quality of the WHP quality criteria be evaluated over time? (2) How does the WHP quality in enterprises change over time and are there typical developments? (3) What role is played by company parameters and implementation phases for the quality of WHP and how it develops over time?

This paper investigates the measurement structure and quality of the quality criteria for WHP dynamically and longitudinally in relation to developments over time and analyses their dependence on selected influencing factors. Firstly, it is necessary to lay down theoretical and methodological foundations for the quality of WHP under aspects of dynamic variability over time and to derive research hypotheses. These are then tested with empirical data from the Austrian QM system for WHP dating from 2014 to 2021 using univariate and multivariate quantitative methods. Finally, the main results are discussed in relation to possible implications for enterprises and the continued development of the quality assurance or QM system.

## Study design and research methods

### Aspects of WHP quality: theoretical and methodological foundations and hypotheses

The theoretical and methodological foundations were based on preliminary considerations in Lang et al. (2019) which regard the quality of WHP as a theoretical construct consisting of dimensions or aspects of quality. Accordingly, the extent of the overall quality of WHP depends on the extent to which individual criteria are fulfilled in relation to the quality of the structure, process, outcome and concept of WHP. On this basis, to specify the theoretical concept of WHP quality, a latent factor was assumed, measured using a multi-attributive instrument reflecting various quality indicators. Although the theoretical concept consists of various components and different properties, the authors assumed that the overall quality of WHP is unidimensional and were able to validate this assumption using confirmatory factor analyses with cross-sectional data (ibid.).

To address the research questions focusing on changes over time and causal relationships between aspects of WHP quality, it was necessary to measure the same quality indicators in the same enterprises at several points in time. To represent the process of change in the collected data and allow for extended validation, a longitudinal measurement and structural model was specified. Confirmatory factor analyses and structural analyses were carried out to investigate both the covariation and stability of inter-individual differences over time. These methods simultaneously allow a structural and dynamic analysis (Geiser 2021), i.e. one which is time dependent.

A longitudinal confirmatory factor model was used with the aim of examining the construct validity and reliability of the measurement model across several measurement points. By introducing restrictions, it was possible to test whether the variability of the factors, the factor loadings and measurement error proved to be invariant quantities over time. To specify the confirmatory factor model longitudinally, it was necessary to measure the quality of WHP as a latent construct that is determined at each point in time across the identical set of indicators (*measurement* or *correspondence hypothesis*). Via the restrictions in the model, it was postulated that the measurement quality of the measured variables does not change over time (Little 2013).

In addition, structural equation modelling (SEM) with longitudinal data was used to examine the assumption of stability or change (i.e. instability) in the quality of WHP and its measurement indicators (*stability hypothesis*). Because we were interested in the true stability of or change in the quality of WHP, a simultaneous analysis of the measurement and structural model with longitudinal data was required. Estimating stable features between WHP qualities on a structural level across several measurement points was only possible once different aspects of reliability and validity had been taken into account. The literature suggests using latent autoregressive SEM (with a so-called Markov structure) in which the quality of WHP is defined as a time-varying latent variable across several panel waves (Geiser 2013). To check for predictive validity, it was assumed that the quality of WHP quality would change between all measurement points, with the actual degree of change only depending on the immediately preceding point in time (so-called first-order autoregressive SEM) but not on its preceding points in time (Reinecke 2005). In addition further time-invariant exogenous variables served to explore concurrent validity, with structural or process-related factors coming into question for the degree of or change in the quality of WHP over time (*structural or process-related hypothesis*).

### Evaluation process and data basis

In the Austrian QM system for WHP, after launching WHP in the company in the form of a WHP pilot project, enterprises can apply for a WHP quality certificate for the first time (the so-called initial award). An application is submitted using a standardized form which provides a comprehensive description of the WHP project. WHP project managers or those responsible submit the filled in application which is signed by the management and also by the workers’ council (if available). The process of being assessed or the quality certificate are free of charge. The applications are passed on to specially trained external experts who examine them independently and evaluate them on the basis of 15 quality criteria (cf. Table 1). The evaluation process makes use of an inspection catalogue to measure the quality of WHP^1^. Using a content-analytic procedure, the individual criteria are evaluated on a uniform rating scale (0 = ‘quality criterion not fulfilled’ to 3 = ‘quality criterion completely fulfilled’)^2^. If a sufficient number of points are obtained across all quality criteria, enterprises are awarded the WHP quality certificate for three years; by going through the same process again, they can apply for re-certification (the so-called renewal award) (Heigl 2017; Lang et al. 2019).

The evaluations analysed in this paper were taken from the WHP QM system and date between 2014 and 2021. In this period, it was possible to identify 570 enterprises with two evaluations and 279 enterprises with three evaluations. The enterprises were located in all four regions of Austria and in all nine federal states. Out of the companies with two evaluations, small and large enterprises were represented almost equally (51.4 vs. 48.6%); in the case of three evaluations, there were fewer small enterprises than large ones (36.9 vs. 63.1%). Just over three quarters of the enterprises with two evaluations (77.5%) had introduced WHP during the observation period of 2014 to 2021 (starting with the initial award of the quality certificate). Enterprises with three evaluations were more evenly divided up between those starting with the initial award (52.0%) and those who were in the sustainability phase (48.0%). The vast majority (> 92%) of the enterprises under consideration had gone through the re-certification process every three years to obtain a new quality certificate, i.e. there were re-evaluations of the quality of WHP for the renewal award/s (cf. Table 2).

**Table 2:** Description of cases

		2 evaluations		3 evaluations	
Variable	Category	n	%	n	%
Total number		570	100.0	279	100.0
Region (federal states)^1^	Eastern Austria (B, NÖ, W)	262	46.0	123	44.1
	Western Austria (OÖ, S, T, V)	216	37.9	111	39.8
	Southern Austria (K, ST)	92	16.1	45	16.1
Size of enterprise	Up to 49 employees	293	51.4	103	36.9
	50 employees or more	277	48.6	176	63.1
WHP phases	In the initial phase	442^2^	77.5	145^3^	52.0
	In the sustainability phase	128^4^	22.5	134^4^	48.0
Time between evaluations	After 3 years	527	92.5	261	93.5
	More than 3 years	43	7.5	18	6.5

^1^*B* Burgenland, *NÖ* Lower Austria, *W* Vienna, *OÖ* Upper Austria, *S* Salzburg, *T* Tyrol, *V* Vorarlberg, *K* Carinthia, *ST* Styria; ^2^ incl. evaluations as part of the initial and 1st renewal award of the WHP quality certificate, ^3^ incl. evaluations for the initial, 1st and 2nd renewal awards; ^4^ incl. evaluations for a series of renewal awards, e.g. 2nd and 3rd (and 4th) renewal awards

### Statistical data analysis

To investigate the longitudinal measurement structure of evaluations of the quality of WHP, repeated measurements were taken at two or three different points (so-called 2- or 3-wave models [2W or 3W] taken at the points t_0_, t_1_ = t_0+1_, t_2_ = t_0+2_) and several confirmatory factor analyses (CFA) were carried out followed by SEM to analyse causality in Mplus 7 (Muthén and Muthén 2012). The theoretical preliminary considerations and derived hypotheses were tested step by step for each of the models.

In a multiple CFA approach in which the quality of WHP is measured as a latent factor at every point in time with 15 indicators (q1–15 for t_0_, r1–15 for t_1_ and s1–15 for t_2_), the first step tested simultaneously for configural measurement invariance and quality (Baseline model 1). Because indicators in longitudinal studies were re-measured at various points in time, modification indices can help identify measurement error specifications (so-called indicator-specific effects) (Geiser 2021; Little 2013; Reinecke 2005). These were specified via covariances between all error variables (so-called autocorrelating residuals) assigned to items with the same content across the measurement points (model 2a). By eliminating non-significant error covariances, model parsimony was ensured (model 2b). In addition, plausible time-specific measurement error covariances could be identified between indicators within one measurement point (Model 2c).

When metric measurement invariance exists between the measurement points, a significant factor correlation is to be taken as indicating an autoregressive model on an error-corrected/structural level (Geiser 2013). This measurement invariance was realized by specifying equality constraints on identical factor loadings over time (e.g. λ_q1_ = λ_r1_ = λ_s1_) (model 3). Autoregressive SEM was used to test the stability of the longitudinal quality of WHP, with the change being ascribed to the immediately preceding measurement point (the so-called first-order autoregressive model 4). An estimated regression coefficient of 0 signifies that there is no relationship and +1/–1 indicates maximal (in-)stability of the quality of WHP over time (Urban and Mayerl 2014). Considering the independent predictors as well was used to estimate the effects of ‘size of enterprise’ and ‘WHP phase’ on the quality of WHP over time (model 5).

To identify the models, the highest factor loading was fixed (value 1) at each measurement point (Note: here indicator 15, representing a general evaluation of WHP activities). For the parameter estimates, the Weighted Least Squares Means and Variance adjusted (WLSMV) method was employed to ensure that the factor loadings and standard errors for indicators with few categories (here: 1 ‘quality criterion not/not really fulfilled’ – 3 ‘completely fulfilled’) can be estimated with less distortion and greater accuracy when a sufficiently large number of cases (as is the case here with n_2W_ = 570, n_3W_ = 279) and a normally distributed latent dimension can be assumed (Cheng-Hsien 2016; Rhemtulla et al. 2012). To evaluate each of the models, standardized and statistically significant effect sizes (*p* < 0.05) were desired that contributed significantly to the factor (factor loadings λ ≥ 0.30) (Bowling 2017). To determine accuracy, Cronbach’s alpha (α > 0.70) was used to establish the reliability of the indicators (Nunnally and Bernstein 1994). The overall fit of the model was determined by the following goodness of fit indices: the χ^2^ test (χ^2^/df < 3.0), the root mean standard error of approximation (RMSEA < 0.08) and the comparative fit index (CFI > 0.90) (Browne and Cudeck 1993; Hu and Bentler 1999; Kline 2011). When testing for measurement invariance, a difference of CFI > 0.01 between two nested models was taken to indicate an absence of measurement equivalence (Cheung and Rensvold 2002). Modification indices served to verify plausible changes to the model (Byrne 2012).

To describe the overall quality of WHP, the 15 indicators were added together at every measurement point to create an overall scale (theoretical range of values: 0–45). It was expected that the variables would have a significant dispersion around the mean (m), i.e. that they would differentiate, and that the distribution of the indicators would not be skewed (s^3^ < |2.0|) and would be unimodal (s^4^ = |7.0|) (Byrne 2012), i.e. there would be no floor or ceiling effects (Döring and Bortz 2016). Taking account of the size of an enterprise (0 = <50 employees vs. 1 = 50+ employees) and the WHP phase it was in (0 = initial phase vs. 1 = sustainability phase), the development of the WHP quality scores over time was tested at three measurement points for significant differences (Wilks λ, *p* < 0.050) with univariate and multivariate analyses of variance (ANOVA, MANOVA) and described using the mean (m) and standard deviation (s).

Homogeneous groups of enterprises with similar developmental patterns in the quality of WHP were identified using cluster analysis (SPSS procedure: quick cluster, algorithm: k-means clustering, max. iterations: 100, convergence criterium: 0.001) and described. To formally determine the optimal number of clusters, solutions were calculated with 1 to 10 clusters and then assessed using statistical criteria (ETA, PRE and FMAX). The preferred cluster solution should be both interpretable in terms of its content (i.e. plausible and consistent) and also have high agreement (κ > 0.75) (Fleiss et al. 2003) with a clustering obtained with randomly chosen initial values; it should also demonstrate the validity of the criteria ‘size of enterprise’ and ‘WHP phase’ (χ^2^ value, *p* < 0.05) (Bacher 2001; Schendera 2010).

## Results

### Measurement quality over time

**Fig. 1:**
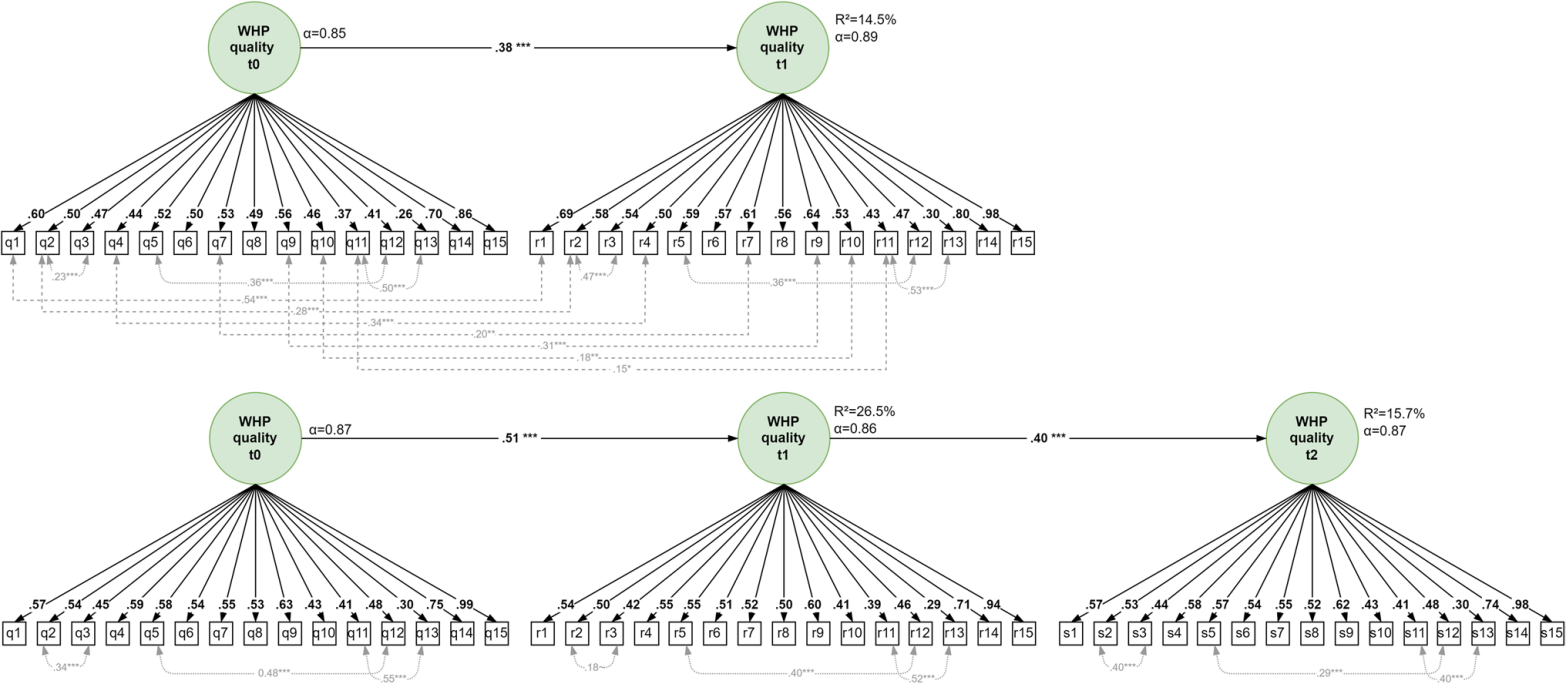
WHP quality using longitudinal data – autoregressive SEM with 2 and 3 measurement points. Notes: 1. Sample: n = 570 for the 2-wave model (top) and n = 279 for the 3-wave model (bottom). 2. The measurement models are metrically invariant with 15 indicators each and q, r and s representing the measurement or evaluation points t_0_, t_1_ and t_2_. 3. Directional arrows indicate standardized factor loadings on the measurement level or stability coefficients on a structural level; bi-directional arrows represent (standardized) measurement error correlations within or between the measurement points. (Note: Due to a lack of space, 21 specified autocovariances between items across the measurement points [q-r-s1, q-r-s2, q-r-s4, q-r-s7, q-r-s9, q-r-s10, q-r-s11] are not shown.). 4. Numerical values represent completely standardized values; estimation method: WLSMV, ***p < 0.001, **p < 0.010, *p < 0.050, ~*p* < 0.080; all factor loadings *p* < 0.001. 5. Model fit for 2 waves: χ²/df = 1.94 | RMSEA = 0.04 (0.036–0.045) | CFI = 0.94. Model fit for 3 waves: χ²/df = 1.32 | RMSEA = 0.03 (0.028–0.039) | CFI = 0.92

Baseline model 1 was characterized by a multiple configural CFA factor model without any restrictions whatsoever, with each factor measuring the quality of WHP at every measurement point with 15 quality indicators. The model fit indicated a good ratio of χ^2^ to degrees of freedom (χ^2^/df_2w_ = 2.73, χ^2^/df_3w_ = 1.46) and a good RMSEA (0.06, 0.04) while the CFI did not indicate an acceptable goodness of fit (CFI_2w_ = 0.88, CFI_3w_ = 0.86) (cf. Table 2). Because the indicators were measured repeatedly, model 2a took account of indicator-specific (method) effects across the measurement points. The covariances between the error variables assigned to the same indicator (so-called autocovariances, for example, between indicators q1-r1 or q1-r1-s1) led to a significant improvement in the model. Non-significant autocorrelations were eliminated again due to better model parsimony in model 2b, which resulted in a slightly improved model fit. Overall, in the 2-wave and 3-wave models, 7 and 21 significant autocovariances (*p* < 0.050), respectively, were specified between items across the measurement points (cf. Fig. 1). Analogous to the results in Lang et al. (2019), the introduction of six significant time-specific measurement error covariances between indicators within one measurement point in the 2-wave model and nine such covariances in the 3-wave model led to a clear improvement in the model. The global fit of model 2c could thus be described as good to excellent (2W/3W: χ^2^/df = 1.79/1.32, RMSEA = 0.04/0.03, CFI = 0.95/0.92), indicating an unchanged factor structure over time or a configural factor and measurement model. In addition, there were significant correlations between the quality factors of WHP across measurement points (2W: r_t0–t1_ = 0.39; 3W: r_t0–t1_ = 0.48, r_t1–t2_ = 0.36 and r_t0–t2_ = 0.29; all *p* < 0.001), which suggests an autoregressive model structure (Table 3).

**Table 3:** Comparison of models

Model	χ^2^/df	RMSEA	90% CI		CFI
			low	high	
2-wave models					
CFA 1: multiple CFA (baseline model)	2.73	0.06	0.051	0.059	0.88
CFA 2a: all autocovariances estimated	2.59	0.05	0.049	0.057	0.90
CFA 2b: only significant autocovariances	2.55	0.05	0.048	0.056	0.90
CFA 2c: incl. time-specific covariances	1.79	0.04	0.033	0.042	0.95
CFA 3: equal factor loadings over t	1.94	0.04	0.036	0.045	0.94
SEM 4: autoregressive SEM (first order)	1.94	0.04	0.036	0.045	0.94
SEM 5: autoregressive SEM with predictors	2.30	0.05	0.044	0.051	0.91
3-wave models					
CFA 1: multiple CFA (baseline model)	1.53	0.04	0.039	0.048	0.86
CFA 2a: all autocovariances estimated	1.46	0.04	0.036	0.045	0.89
CFA 2b: only significant autocovariances	1.46	0.04	0.036	0.045	0.88
CFA 2c: incl. time-specific covariances	1.32	0.03	0.028	0.039	0.92
CFA 3: equal factor loadings over t	1.32	0.03	0.028	0.039	0.92
SEM 4: autoregressive SEM (first order)	1.32	0.03	0.028	0.039	0.92
SEM 5: autoregressive SEM with predictors	1.39	0.04	0.032	0.042	0.90

*CFA* confirmatory factor analysis, *SEM* structural equation model, *χ*^*2*^ chi-square test statistic, *df* degrees of freedom, *RMSEA* root mean standard error of approximation, *CI* confidence interval, *CFI* comparative fit index

### Changes in WHP quality over time

The factor loadings on identical indicators across the measurement points were equated in model 3 using restrictions, which resulted in an acceptable or only marginally worse CFI (ΔCFI_2w_ = –0.01, ΔCFI_3w_ = –0.001), thus confirming the presence of metric measurement invariance. The metrically invariant 2-wave and 3-wave models had average standardized factor loadings of λ_m_ = 0.51 and 0.56, respectively. Only indicator 13 in the 2- and 3-wave models fell below the required minimum (2W λ_q13_ = 0.26; 3W λ_q13_ = 0.29) due to the relatively high standardized measurement error correlation of r = 0.50 and 0.53 (*p* < 0.001), respectively, between indicators 11 and 13. Cronbach’s alpha coefficients were satisfactory for all measurement points in both types of models (2W α = 0.85 and 0.89; 3W α = 0.87, 0.86 and 0.87).

The first-order autoregressive SEM in model 4 with metric measurement invariance estimated the stability of the quality of WHP across the measurement points. In the 2-wave model the regression coefficient was β_t0–t1_ = 0.38 with an explained variance of R^2^_t1_ = 14.5% and in the 3-wave model the regression coefficient was β_t0–t1_ = 0.51 with R^2^_t1_ = 26.5% and β_t1–t2_ = 0.40 with R^2^_t2_ = 15.7% (*p* < 0.001). The modification indices in the 3-wave model did not demonstrate a significant effect from the first to the third measurement point, thus implying no second-order autoregressive structure (cf. Fig. 1).

**Fig. 2:**
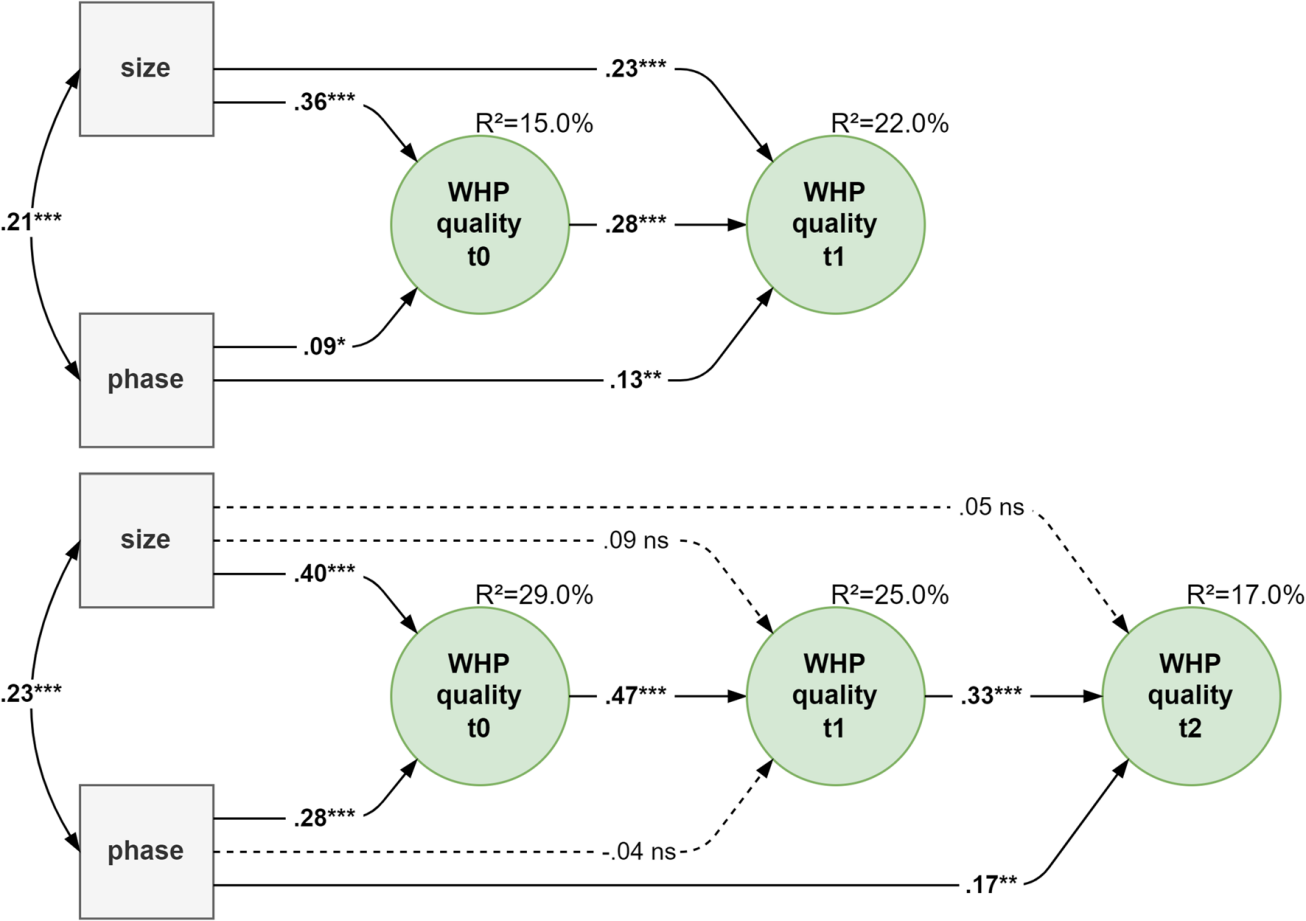
WHP quality over time with predictors. Notes: 1. Sample: n = 570 for the 2-wave model (top) and n = 279 for the 3-wave model (bottom). 2. Metrically invariant measurement models with 15 indicators each are not shown due to a lack of space. 3. Size of the enterprise: 0 = <50 employees, 1 = 50+ employees; phase of WHP: 0 = initial phase, 1 = sustainability phase. 4. Numerical values: completely standardized values, estimation method: WLSMV, ***p < 0.001, **p < 0.010, *p < 0.050, ns = not significant. 5. Model fit: 2-wave model: χ²/df = 2.30 | RMSEA = 0.05 (0.044–0.051) | CFI = 0.91. 3-wave model: χ²/df = 1.39 | RMSEA=0.04 (0.032-0.042) | CFI = 0.90

Model 5 took account of two additional control variables in the autoregressive longitudinal models. In the 2-wave model larger enterprises (γ_t0_ = 0.36 and γ_t1_ = 0.23; both *p* < 0.001) as well as those in the sustainability phase (γ_t0_ = 0.09 and γ_t1_ = 0.13; both *p* < 0.050) had a significantly higher quality of WHP at both the first and second evaluation or measurement points. In the 3-wave model, both variables were influential as well, but the size of the enterprise was only so for the first evaluation or measurement point (γ_t0_ = 0.40, *p* < 0.001) and the WHP phase for the first (γ_t0_ = 0.28, *p* < 0.001) and third points (γ_t0_ = 0.17, *p* < 0.010). The size of an enterprise and the phase correlated weakly at r_2w_ = 0.21 and r_3w_ = 0.23, respectively (*p* < 0.001). Compared with model 4, the stability coefficients had similar values (2W: β = 0.28; 3W: β = 0.47 and 0.33, respectively; all *p* < 0.001) and explained a progressively decreasing but relevant proportion of variance from 15 to 29% (cf. Fig. 2).

### Influence of company parameters on the quality of WHP, developments over time

The means and dispersion of the WHP overall quality score only varied marginally across the three measurement points from m_t0_ = 35.3 (s_t0_ = 4.9) and m_t1_ = 36.7 (s_t1_ = 4.3) to m_w2_ = 35.7 (s_t2_ = 4.3), although the mean at t_1_ (reference value) is significantly different in comparison with those at t_0_ and t_2_ (t_t0_ = –4.75, t_t2_ = –3.85, df = 278, *p* < 0.001). The quality of WHP was mildly skewed (s^3^_t0_ = 0.10, s^3^_t1_ = –0.36, s^3^_t2_ = –0.19) with an almost unimodal distribution (kurtosis: s^4^_t0_ = –0.52, s^4^_t1_ = –0.16, s^4^_t2_ = –0.43) (cf. Table 4).

**Table 4:** Comparison of WHP quality over measurement points

Variable	n	t_0_			t_1_			t_2_		
		m	s	95% CI	m	s	95% CI	m	s	95% CI
Total number	279	35.3	4.9	34.7–35.9	36.7	4.3	36.2–37.2	35.7	4.3	35.2–36.3
Size of enterprise										
Small	103	32.7	3.9	31.9–33.5	35.4	4.1	34.6–36.2	35.2	4.4	34.3–36.1
Large	176	36.9	4.8	36.2–37.6	37.5	4.2	36.9–38.2	36.1	4.3	35.4–36.7
Phase										
Initial	145	33.9	4.5	33.1–34.6	36.2	4.4	35.5–36.9	35.1	4.6	34.3–35.8
Sustainability	134	36.9	4.9	36.1–37.8	37.3	4.1	36.6–38.0	36.5	3.9	35.8–37.2
Cluster										
Decrease	98	40.5	2.6	40.0–41.0	39.9	3.0	39.3–40.5	36.8	3.7	36.0–37.5
Increase	98	33.2	2.7	32.6–33.7	36.8	3.4	36.1–37.5	38.4	2.9	37.8–39.0
Stable	83	31.8	3.9	30.9–32.6	33.0	3.6	32.2–33.8	31.4	2.9	30.8–32.0

*n* valid cases, *t*_*0*_*, t*_*1*_*, t*_*2*_ measurement points, *m* mean, *s* standard deviation, *CI* confidence interval

The average WHP overall quality score depended on both the size of an enterprise and the phase it was in. The differences were greatest at the first measurement point, with small enterprises and enterprises from the initial phase onwards (initial award and 1st renewal award) being able to improve the most. All means have in common that there was a slightly lower mean WHP overall quality score at the third measurement point than at the second measurement point, although the dispersion around the mean for large enterprises and those in the sustainability phase decreased over time, while that of small enterprises and those in the initial phase of WHP increased.

The results derived from the MANOVA test^3^ make it clear that the size of an enterprise (Wilks λ = 0.86, F[3, 273] = 15.08, *p* < 0.001) and the WHP phase (Wilks λ = 0.94, F[3, 273] = 5.388, *p* < 0.010) had a significant multivariate correlation with the quality of WHP at all three measurement points. The ANOVAs for each measurement point revealed that the size of an enterprise and the quality of WHP correlated at the first (F[1, 278] = 42.40, *p* < 0.001) and second measurement point (F[1, 278] = 14.03, *p* < 0.001) but not at the third (F[1, 278] = 1.06, *p* = 0.305) and that the WHP phase correlated with the first (F[1, 278] = 13.35, *p* < 0.001) and third measurement point (F[1, 278] = 4.96, *p* < 0.050) but not with the second (F[1, 278] = 1.27, *p* = 0.260).

### Types of WHP quality change

A comparison of the 10 cluster solutions suggested that k = 3 clusters was the best solution: firstly, because the explained dispersion up to the 3-cluster solution increased every time by 13% in absolute terms (ETA_k = 3_ = 0.50); secondly, because the PRE coefficients up to three clusters showed a relative improvement of over 20% in comparison with the previous solution; and thirdly, because although the FMAX criterion was highest for two clusters, it was still relatively high for three clusters before falling continuously after that. Moreover, the solution with three clusters was very stable because it matched strongly with randomly generated starting values (κ = 0.98).

The three identified clusters can be described and interpreted as follows. Cluster 1 was characterized by a drop in the quality of WHP over time with the mean score decreasing steadily from a high level and dropping particularly at the third measurement point (m_t0_ = 40.5, m_t1_ = 39.9, m_t2_ = 36.8). This cluster comprised n = 98 (35.1%) of the 279 enterprises which were analysed. Cluster 2 in contrast was characterized by a steadily increasing quality of WHP (m_t0_ = 33.2, m_t1_ = 36.8, m_t2_ = 38.4), starting out at a lower level and flattening out over time. Cluster 2 consisted of another n = 98 enterprises (35.1%). The centroids of WHP quality in cluster 3 ranged from m_t0_ = 31.8 via m_t1_ = 33.0 to m_t3_ = 31.4; in other words, they hardly changed in absolute terms and can therefore be described as ‘stable’. A fairly constant quality of WHP was observed in n = 83 enterprises (29.7%). In comparison with the reference measurements (here t_1_) of each cluster, the centroids had a statistically significant difference.

In the period under study from 2014 to 2021, 42.7% of the smaller enterprises were able to increase the quality of WHP, 42.7% could keep it roughly stable and in 14.6% of the enterprises, the quality of WHP decreased. In comparison to the larger enterprises where 30.7% showed a gain, 22.2% kept the quality stable and 47.2% showed a loss in quality over time (χ^2^ = 31.57, df = 2, *p* < 0.001). In enterprises which were in the initial phase of WHP in the period under study, 36.6% were able to increase it, 41.4% kept the quality stable and in 22.1% of the enterprises, the quality fell. In the sustainability phase, quality improved for 33.6% enterprises, stayed the same for 17.2% and decreased over time for 49.3% of the enterprises (χ^2^ = 28.55, df = 2, *p* < 0.001).

## Discussion

This paper aims to (1) examine the measurement quality of the WHP quality criteria and the quality of WHP in enterprises over time (*correspondence hypothesis*), (2) observe changes in the WHP quality in enterprises and potentially typical developments (*stability hypothesis*) and (3) test company parameters and implementation phases for the quality of WHP and how it develops over time (*structural or process-related hypothesis*).

### Research question 1

The results proved that the concept of 15 quality criteria can be used to observe and evaluate the quality of WHP in enterprises in a valid and reliable manner, both cross-sectionally and longitudinally (at two and three observation points/waves). The measurement proved to be empirically successful across several measurement points in identical enterprises and the use of the 15 quality criteria allowed a high-quality assessment of the quality of WHP. On the one hand, it was possible to confirm the time-invariant validity of the measurement models due to the verified construct and criterion validity of the measurements over time. On the other hand, the quality criteria used were suitable for a reliable longitudinal observation of the quality of WHP because the survey and evaluation process ensured not only consistent and equivalent, i.e. equal and invariant, measurements but also relative stability, i.e. the equality or similarity of the measurement results (internal consistency and test-retest reliability) when used at varying measurement points.

Alongside time-related random measurement errors, time-stable error components were also identified in the quality indicators, where unobserved variables not included in the model could be responsible for the residual correlations, for example (Reinecke 2005). The need to specify equal measurement error covariances across the different measurement points is due to the repeated measurement process. In contrast, the measurement error covariances within each point in the survey indicated or replicated already known (but negligible) measuring problems that are rooted in the dimensions of the quality of the structure, process, outcomes and concept relevant for WHP and which can be analysed with the aid of method- or bi-factors, for example (cf. Lang et al. 2019).

### Research question 2

At the level of quality in the enterprises, the results were stable. The successful empirical proof of the factorial invariance of the measurement (here both configural and metric measurement invariance) was a necessary condition to be able to compare and explore the changes of the quality of WHP in enterprises over time (see Little 2013). The quality of WHP was not subject to specific measurement fluctuations and had stable properties over time. Our results suggest that the quality of WHP remained relatively stable in the enterprises in our study over a period of approx. 12 years, the emphasis being on relatively stable as this relates to the mean across all enterprises. The explorative cluster analysis revealed three typical, interpretable and plausible yet different change patterns over time. In around one third of the enterprises, the quality remained similarly high and stable over time, in another third the quality could be improved (from a low starting point) and in the last third of the enterprises the quality deteriorated over time.

### Research question 3

Here we used longitudinal data to empirically replicate something that Rojatz et al. (2017) pointed out in their review: the realization of WHP quality and its observed development over a longer period of time is (partially) determined by structural parameters and conditions of implementation which affect the process within the enterprise. Bearing in mind that the ‘resources’ available to companies vary greatly, the structural determinant was related to the size of an enterprise (smaller vs. larger enterprises). The implementation process, in turn, was broadly represented by the extent to which WHP was anchored in the enterprise or rather the phase of WHP in the company, i.e. the initial or sustainability phase (maturity of WHP, implementation of workplace health management). For the purpose of concurrent and predictive validity, the two proxy variables were responsible for both the level of quality at the measurement point in question and the changes in quality over time. In more concrete terms, in comparison with larger enterprises or those in the sustainability phase of WHP, smaller enterprises or enterprises in the initial phase of WHP achieved lower levels of WHP quality, something which is also picked up on in reviews of the literature (Hollederer and Wießner 2015; McCoy et al. 2014). The documented effects of ‘size of enterprise’ and ‘WHP phase’ also declined in importance over time, which is presumably related to further influencing factors gaining in importance: internal project managers could have improved their competences via a consultant/client system, for example (Bauer et al. 2014) and the quality certificate itself should boost both organization and management.

WHP over time was dependent on the structure, process, outcome and (quality of the) concept. Effective WHP is contingent upon many factors and sustainability can only be achieved in a longer-term implementation phase. Successful and sustainable WHP is achieved through a combination of facilitating and hindering structural (framework) conditions in a process involving preparation, needs assessment, planning and implementing measures and evaluation. Once introduced, the quality of WHP becomes a stable property of companies when they manage to set up permanent high-quality structures and processes based on a good concept, when WHP is lived by the management and when it becomes part of corporate culture (Badura et al. 2016; Schein 2010). One comprehensive review came to the conclusion that there are significant correlations between culture of health elements and the health and safety of employees (Flynn et al. 2018). The size of an enterprise and WHP phase affect changes in the quality of WHP over time (recurring implementation cycles). This is in line with expectations but also indicates that small enterprises should be motivated and supported even more noticeably in relation to their WHP measures. Tailor-made support services when implementing WHP but particularly in the sustainability phase as well are essential as not all enterprises can maintain quality in the long term and run the risk of the effect of WHP being reduced or even disappearing because quality criteria are only fulfilled to a limited extent, for example.

## Methodological limitations

In the extended investigation, numerous aspects of validity and reliability were confirmed, although the chosen research approach had several methodological and content-related issues which are worth mentioning. The study made use of a cross-sequential design that followed up on an cohort of enterprises over several years and was particularly well suited to investigating processes of change and controlling for possible differences between the cohorts. One weak point is that there are possible confounding interactions between the cohorts and measurement points (Little 2013). Several aspects can jeopardize the validity of longitudinal studies, although instrumentation effects due to changed measurement characteristics over time can be excluded on the basis of the factorial invariance of the measurement instrument and in this light no restrictions are to be expected in the significance of changes over time. A regression to the mean due to poor reliability can also be excluded on the basis of confirmatory factor analysis, i.e. changes over time are captured well. A test–retest effect cannot be completely excluded, in contrast, as multiple applications and evaluations for the WHP quality certificate cannot be independent of previous measurements (e.g. recognizable by the homogenization effect with decreasing variance over time). Also, recall the fact that the enterprises in the study are not a representative cross-section and there could be a selection effect to the extent that mostly enterprises with sufficient WHP quality submit an application for (re-)certification (cf. the proportion of larger and smaller enterprises with two and three evaluations). The intention of the present study was to trace changes in the quality of WHP in enterprises over time, which only works with so-called prototypical enterprises (Diamantopoulos 2005) that were recorded in the QM system over a longer period of time. Selective attrition is not unlikely when enterprises which had been recorded did not apply for a renewal award for the WHP quality certificate, i.e. panel mortality does not occur purely by chance.

In line with this point of a “selection bias”, it could be argued that especially smaller enterprises have only small resources for WHP and this could limit our results. This is true and structural factors such as size can affect several aspects as discussed in the introduction (see Hollederer and Wießner 2015; McCoy et al. 2014; Taylor et al. 2016). This is a principal aspect, but it does not limit the results of our study as we focus on the enterprises which undergo the process of WHP quality assessment. Therefore, the results cannot be transferred to all companies which investigate WHP but we can see positive and critical factors affecting the motivation for WHP programmes in principle.

Also, it is important to stress that the quality criteria themselves do not give an evaluation of the health improvements; therefore, this aspect is not in the focus. Future research could investigate the relationship of WHP quality criteria/assessments with health measures (e.g. absenteeism, presentism, work burdens, work satisfaction, etc.).

Nonetheless, to the best of our knowledge, this study is the first longitudinal investigation of WHP quality using these data and methods. Most longitudinal studies only cover a limited period of time; here developments relating to quality could be traced over approx. 12 years. More than three measurement points would reveal even deeper insights. Further gains in knowledge would be achieved by expanding the Markov model with more time-varying (or time-invariant) factors or constructs in order to explore concurrent validity and relationships between WHP quality or developments relating to quality and external criteria (work-related resources and burdens, presenteeism, employee satisfaction, exercise and eating habits, state of health and well-being, etc.). It would also be advantageous to use more advanced methods of analysis such as latent growth models (Geiser 2021; Reinecke 2005).

The analysis at hand traced the importance of the size of an enterprise and the WHP phase, two relevant factors in the establishment and stabilization of WHP in a company. As this can only represent a rough approximation of relevant structures and processes, future studies should take account of or control for further determinants of WHP quality and the developments it undergoes in their analysis (cf. Rojatz et al. 2017).

## Conclusions

One of the functions of the Austrian QM system is to define and safeguard the quality of WHP based on solid scientific principles. This includes an assessment of whether it meets the requirements in theory and practice. Research into WHP is therefore interested in studying changes over time and linking such changes with other variables as well as attempting to disentangle and separate causes and effects (Geiser 2021). The question of (typical) patterns of change in the quality of WHP in enterprises was investigated, including whether there are determining factors (predictors, covariates) in corporate structures and processes which influence social patterns of change or individual changes. The results presented here contribute to an extended validation of WHP because they cover in-depth insights into the stabilization and sustainability of WHP processes and structures in companies for the first time. Overall, they reveal completely new findings on developments relating to the quality of WHP and under which conditions and in which phase (initial or sustainability phase) these can be achieved in a company, including empirically robust statements on the (reciprocal) effect of WHP quality und relevant (external) criteria. The results presented in this article make an important contribution to the state and variability of social structures and systems in WHP, answering the question as to how enterprises develop WHP and (can) put it on a permanent footing so that they can achieve a sustainable effect in WHP.

In this respect, the findings serve to advance WHP and its quality assurance in general. That is not only valuable for evaluating WHP but also for monitoring or managing it. Here the relevant questions include: What do enterprises need for this? What implications do these have for enterprises? In any case, enterprises need support systems to maintain the quality of WHP on a permanent basis. In addition, WHP must move with the times and adapt to changing conditions and challenges in the world of work (e.g. digitalization of work, an ageing workforce, economic and ecological crises). These insights should be discussed with the aim of advancing quality assurance in WHP and deployed in public health.

## Data Availability

Not applicable.

## Footnotes

1 As employee assessments are an integral part of the WHP process, they are also included in the quality assessment, e.g. in specific quality criteria such as target group and employee orientation or diagnostic phase/tool/needs assessment.

2 A concrete example regarding the quality criterium ‘attainment of goals’: No point is given if no realistic WHP project goals were defined at all. One point if most of the objectives were not achieved or if the achieved objectives are not traceable. Two points if the majority of the objectives have been achieved and are relatively traceable or the non-achievement is traceable or plausible. Three points if the objectives have been well achieved and there is good evidence to support this.

3 Test for multivariate normality: Shapiro–Wilk = 0.99, *p* = 0.104
